# G protein-coupled receptors and traditional Chinese medicine: new thinks for the development of traditional Chinese medicine

**DOI:** 10.1186/s13020-024-00964-4

**Published:** 2024-07-02

**Authors:** Ting Zhang, Wenqiao An, Shengjie You, Shilin Chen, Sanyin Zhang

**Affiliations:** 1 Institute of Herbgenomics, Chengdu University of Traditional Chinese Medicine, Chengdu, 611137 China; 2 Innovative Institute of Chinese Medicine and Pharmacy, Chengdu University of Traditional Chinese Medicine, Chengdu, 611100 China

**Keywords:** G protein-coupled receptors, Traditional Chinese medicine, TCM syndrome, Drug development

## Abstract

G protein-coupled receptors (GPCRs) widely exist in vivo and participate in many physiological processes, thus emerging as important targets for drug development. Approximately 30% of the Food and Drug Administration (FDA)-approved drugs target GPCRs. To date, the ‘one disease, one target, one molecule’ strategy no longer meets the demands of drug development. Meanwhile, small-molecule drugs account for 60% of FDA-approved drugs. Traditional Chinese medicine (TCM) has garnered widespread attention for its unique theoretical system and treatment methods. TCM involves multiple components, targets and pathways. Centered on GPCRs and TCM, this paper discusses the similarities and differences between TCM and GPCRs from the perspectives of syndrome of TCM, the consistency of TCM’s multi-component and multi-target approaches and the potential of GPCRs and TCM in the development of novel drugs. A novel strategy, ‘simultaneous screening of drugs and targets’, was proposed and applied to the study of GPCRs. We combine GPCRs with TCM to facilitate the modernisation of TCM, provide valuable insights into the rational application of TCM and facilitate the research and development of novel drugs. This study offers theoretical support for the modernisation of TCM and introduces novel ideas for development of safe and effective drugs.

## Introduction

G protein-coupled receptors (GPCRs) widely express on the cell membrane, constituting the largest protein family encoded by the human genome [1]. G proteins can bind guanosine triphosphate (GTP) and guanosine diphosphate (GDP) and have three distinct subunits: α, β and γ [2]. They are widely distributed in various tissues and cells of the human body, participating in the regulation of body development and performing various physiological functions [3]. GPCRs are divided into five major families according to the sequence and structural similarity, namely, rhodopsin-like (class A), secretin-like (class B1), glutamate-like (class C), frizzled-like (class F) and adhesion (class B2) receptors ( Table 1). Class A has the largest proportion and the most widely studied [4] and is related to cardiovascular diseases, such as hypertension, lung diseases and mental illnesses, such as depression [5]. Class B1 GPCRs, such as glucagon-like peptide-1 receptor (GLP-1R) and glucagon receptor can regulate glucose homeostasis and lipid metabolism [6–8]. The Class B2 receptors are critical to the regulation of sensory, endocrine and gastrointestinal systems [9]. The physiological function of class C GPCRs has been linked to cancer, migraine, schizophrenia and movement disorders [10]. Class F GPCRs are mainly associated with cancer, fibrosis and embryonic development [11].

**Table 1:** Overview of GPCR subfamilies and their physiological functions

Class	Related disease	Example	Ref.
Rhodopsin (A)	Cardiovascular diseases such as high blood pressure, lung diseases and mental illnesses such as depression	AT1R, AT2R, GPR35,40,41,120, β-adrenergic receptors	[5, 15, 16]
Secretin (B1)	Regulating blood glucose homeostasis and lipid metabolism	GLP-1R, GCGR	[6–8, 17]
Adhesion (B2) (aGPCR)	Modulating sensory, cardiovascular, endocrine, and gastrointestinal systems	GPR56	[9, 18]
Glutamate ©	Cancer, migraine, schizophrenia, and movement disorders	NMDAR, mGluRs	[10, 19, 20]
Frizzled (F)	Cancer, fibrosis, and embryonic development	Fzd5	[11, 21]

*AT1R* angiotensin type 1 receptor, *AT2R* angiotensin type 2 receptor, *GLP-1R* glucagon-like peptide-1 receptor, *GCGR* glucagon receptor, *GPCR35* G protein-coupled receptor 35, *NMDAR* *N*-methyl-d-aspartate-receptor, *mGluRs* group I metabotropic glutamate receptors, *Fzd5* Frizzled Homolog 5, *GPR56* G protein-coupled receptor 56

GPCRs are the key regulators of various pathological processes and drug targets for many diseases [12], and more than 30% of FDA-approved drugs target GPCRs [13]. In 2023, ten drugs approved by the Food and Drug Administration (FDA) are related to GPCRs, which accounted for 18.2% (10/55) of the approved drugs in that year [14]. Given the considerable number of GPCR drugs approved by the FDA that year, the development and clinical application of GPCR drugs is expected to continuously expand rapidly.

Traditional Chinese medicine (TCM) compounds are used for the prevention, treatment and diagnosis of diseases, facilitate rehabilitation and have health benefits [22]. TCM usually involves multiple components, pathways, and targets, synergistically exerting its pharmacological effects [23, 24]. The theoretical framework of TCM emphasises ‘syndrome differentiation and treatment’, that is, a treatment plan is determined according to the specific physique and symptoms of a patient [25]. ‘Syndrome differentiation and treatment’ is related to the diversity of GPCRs, and the expression levels and activities of different GPCRs vary in different tissues and disease states. By studying the correlation between GPCRs and TCM syndromes, we can comprehend the regulatory mechanisms of TCM on individuals. Therefore, combining GPCRs with TCM may contribute to the modernisation of TCM.

**Fig. 1:**
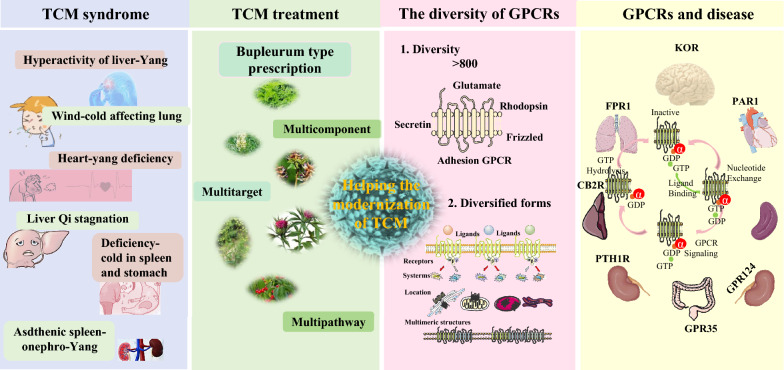
GPCRs and TCM: helping the modernisation of TCM. TCM syndromes are complex diagnostic systems, such as hyperactivity of liver-yang, which is often presented as headache and dizziness. The diversity of multi-component, multi-target, and multi-pathway TCM is consistent with GPCRs, such as KOR, GPR35 and FPR1, which are involved in the regulation of a variety of diseases and are closely related to brain injury, ulcerative colitis, and lung adenocarcinoma, respectively. *CB2* cannabinoid receptor, *FPR1* formyl peptide receptor 1, *GPR35* G protein-coupled receptor 35, insulin-like growth *KOR* kappa opioid receptor, *PAR1* protease-activated receptor 1, *PTH1R* parathyroid hormone type 1 receptor

In this paper, the advantages of the joint study of GPCRs and TCM are reviewed from the aspects of GPCRs and TCM syndromes, the characteristics of multi-component and multi-target TCM and drug screening methods. The aim is to solve problems in TCM in terms of active ingredients, molecular mechanisms, and clinical efficacy (Fig. 1). The concept of ‘Simultaneous screening of drugs and targets can clarify the characteristics and targets of drugs and is in line with the basic principles of TCM. Therefore, the research and development of TCM targeting GPCRs is considered scientific and conducive to the modernisation of TCM and international demand.

## GPCRs and TCM syndromes: elucidating the theoretical basis and biological mechanism of TCM

The syndrome is a term exclusive to TCM and is summarised as a series of interrelated symptoms, that is, the intrinsically organic reaction state of disease location, aetiology, disease nature, disease potential and strength of the body’s disease resistance at a certain stage of the disease process, which are manifested as clinically observable symptoms.

### Individualised diagnosis of TCM syndromes and diversity of GPCRs

A syndrome is a non-linear complex giant system of ‘internal reality and external deficiency’, ‘dynamic space–time’ and ‘multi-dimensional interface’ [26]. Owing to the wide variety of diseases, syndromes are constantly changing, and 2060 TCM syndromes have been identified [27]. Some syndromes are easy to distinguish, and some complex mixed and complex syndromes have been identified. Two-deficiency syndromes include the heart and kidney; heart and lung; heart and spleen; liver and kidney; and spleen and kidney. The clinical manifestations of a syndrome vary by disease. For example, in a kidney yang deficiency syndrome [28], urination can be manifested as clear and long. However, it can also be manifested as short urination or retention of urine. Kidney diseases are mostly deficiency syndromes in TCM, such as children’s nephrotic syndrome and IgA nephropathy in a certain stage of the development of the disease (like the later stage of the disease or recurrence), and can be manifested as kidney yang deficiency with six yin syndromes; clinical manifestations can be weak waist and knee, cold limbs and long urination [29] or lack of urine [30]. The complexity of TCM syndromes is the functional complexity of the same GPCRs in diseases. For example, the expression and function of the same GPCR vary by disease. CB1R is upregulated in liver fibrosis, promoting liver fibrosis [31]. However, CB1R is downregulated in colorectal cancer, and the activation of CB1R can improve rectal cancer [32]. The activation of free fatty acid receptor 4 (FFAR4), also known as G protein-coupled receptor 120, reduces atherosclerosis and protects heart function [33]. However, the activation of the FFAR4 signalling pathway can promote the growth and migration of colon cancer cells [34].

The complexity of TCM syndromes is the functional activity of the GPCR regulatory network to regulate various cell functions. The activation of GPCR regulatory subunits has shown a variety of therapeutic effects [35]. Coupling with different ligands leads to the same transduction pathway and has different relative potency, like TCM ‘same disease with different treatment’. Mogamulizumab, which acts on CXCR4, was approved by the FDA in 2012 for the treatment of relapsed or refractory adult T-cell leukaemia lymphoma [36] and in 2018 for the treatment of cutaneous T-cell lymphoma (Sezary syndrome, granuloma fungoides) [37]. Therefore, the complexity of TCM syndromes and the functional complexity of GPCRs are similar.

### Dynamic changes in syndromes and GPCRs regulation

**Fig. 2:**
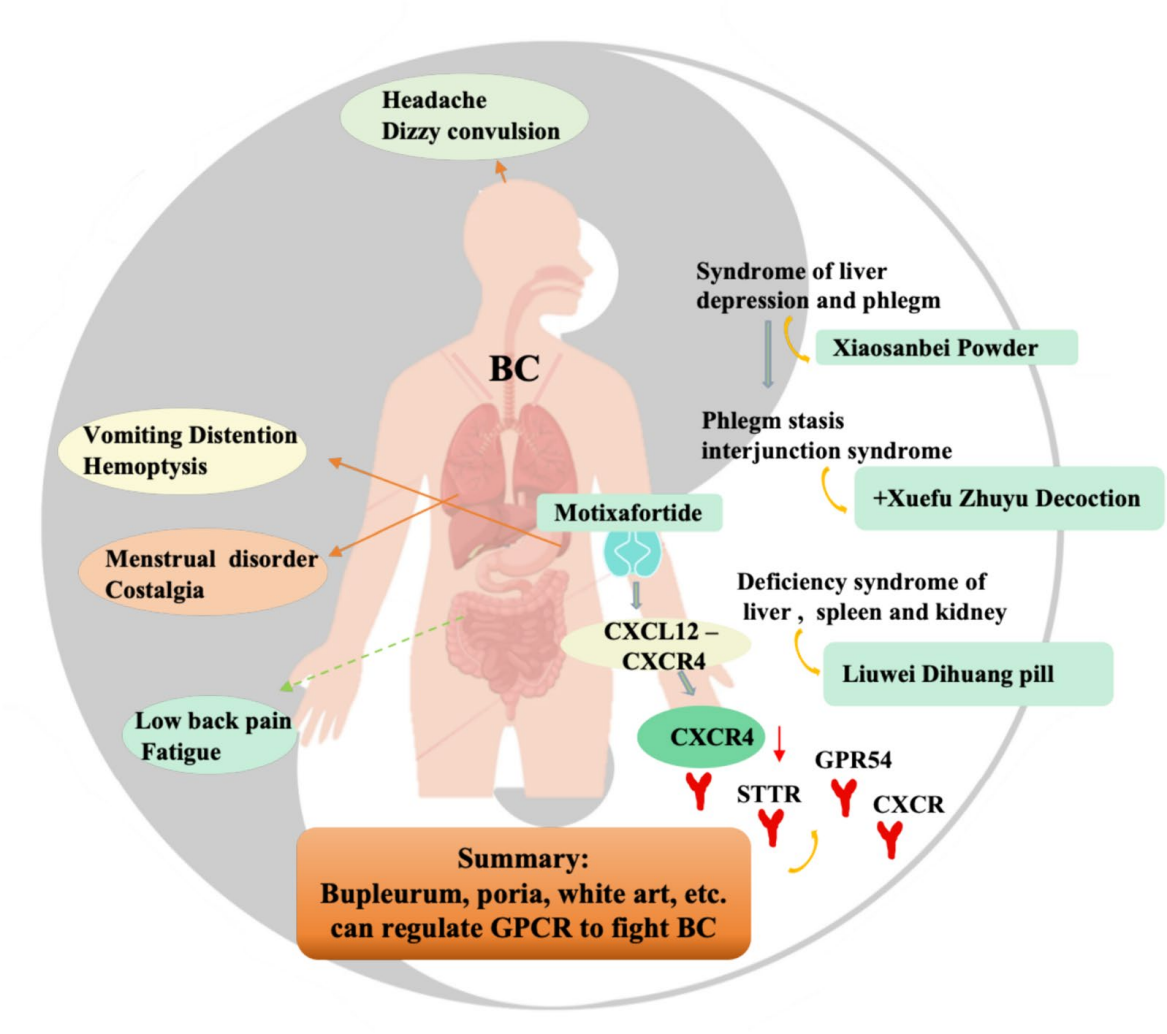
Application of TCM diagnosis and GPCRs in the treatment of breast cancer. As the symptoms of breast cancer change, the composition or dosage of TCM changes. TCM products include *bupleurum*, *poria*, and *white rhizoma*. The FDA-approved anticancer drug motixafortide can regulate GPCRs (CXCR, STTR and GPR54) against BC. *BC* breast cancer, *CXCR* chemokine receptor, *GPR54* G protein-coupled receptor 54, *SSTR* somatostatin receptor

TCM syndromes are dynamic and constantly adjusted as the disease changes [38]. An untreated wind-heat surface syndrome can become an inner-heat syndrome. In an untreated wind-cold superficial syndrome, cold stagnates in the muscle surface, which can turn heat into superficial heat syndrome or become superficial cold inner heat syndrome or inner heat syndrome [39]. Similar to changes in syndromes, the downstream signals of GPCRs vary under different conditions. In the inactive state, the Gα subunit binds to guanine nucleotide GDP. Upon receptor activation, GDP is replaced by GTP, which dissociates the Gα subunit from the βγ dimer, and both subunit complexes promote intracellular effector networks, including the second messenger production system, small GTPase and kinase cascades, such as MAPK and PI3K/Akt, leading to changes in gene transcription and cellular events [40]. In addition to this regulatory process, GPCRs can migrate from the cell surface to the endosome to activate specific genes [41]. In breast cancer, which has the highest incidence among women in the world [42], for example, TCM believes that ‘stagnation of liver qi and chong-ren disorder’ is one of the core causes of breast cancer, and ‘soothing liver, tonifying kidney and promoting blood circulation’ is the most commonly used treatment method for breast cancer (Ruyan) in ancient books from the Sui Dynasty to the Qing Dynasty. During breast cancer, it experienced changes in symptoms such as liver stagnation and phlegm stagnation, late deficiency of the liver and kidney and deficiency of spleen and kidney. Along with the changes in these syndromes [43], the overexpression and abnormal activation of GPCRs participate in the development of breast cancer (Fig. 2).

### Comprehensive analysis of TCM syndrome and GPCR signal network

A syndrome is a key factor in TCM. For the syndrome of liver depression and spittoon coagulation, the liver should be cleared, qi should be regulated, phlegm should be dissolved, and formation should be dispersed. Xiaosanbei powder is added or reduced. For phlegm and blood stasis interjunction syndrome, Xiaosanbei powder combined with Xuefu Zhuyu decoction is used to reduce symptoms, and Angelica, Peach Kernel and Leonurus are added to promote blood circulation and remove blood stasis. For the liver and kidney deficiency syndrome, the Liuwei Dihuang pill combined with Gulu Erxian Dan nourishes the liver, kidneys, and marrow. In the spleen and kidney deficiency syndrome, the spleen and kidney should be strengthened. For this syndrome, Liuwei Dihuang pill combined with Sijunzi decoction can be used to reduce symptoms, along with raw turtle shell, deer horn glue and ejiao and other strong tonic drugs [44]. TCM increase or decrease drugs or change prescriptions with the change of syndrome is similar to the different effects of GPCRs binding to specific ligands in different states [45]. Lipoxin or resolvin can bind to the FPR2 receptor, inhibiting the nuclear factor κB (NF-κB) signalling pathway, upregulating the Nrf2 and peroxisome proliferator-activated receptor γ (PPARγ) signalling pathways and reducing the expression of tumour necrosis factor α (TNF-α) and interleukin 1β (IL-1β) [46, 47]. However, the FPR2 contains NF-κB binding sites. When the pro-inflammatory ligands of FPR2 (such as serum amyloid A peptide) bind to it, the NF-κB signalling pathway is activated, promoting the release of three well-known inflammatory factors: TNFα, IL-1β and IL-6 [48].

*Radix bupleurum*, *Poria Cocos*, *Rhizome*, *Peach Kernel*, *Mountain Mushroom*, *Astragalus* and *Curcuma* can treat breast cancer by inhibiting protease activating receptor 1 [49], chemokine receptor [50, 51], somatostatin receptor and GPR54 [52]. Therefore, the prescription can affect the development of a disease by regulating GPCRs and subtypes. CXCR4 plays a central role in tumour progression, angiogenesis, metastasis and cell survival, and its dysfunction is directly linked to various forms of cancer, often where it is not only overexpressed but also overactivated [50]. Motixafortide is the best CXCR4 antagonist, and all its alkaline residues establish interactions with residues in the CXCR4 orthosteric binding site, which seems to be the driving force behind motixafortide’s high affinity for the CXCR4 receptor [53]. Motixafortide acts on the CXCL12–CXCR4 signal axis [54] and has been used in the preclinical studies of pancreatic, breast and lung cancer [55]. Therefore, we revealed the basic theory and biological mechanism underlying TCM syndromes from the perspective of GPCRs to provide a scientific basis for the promotion of TCM.

## Applying GPCRs in Chinese medicine research is beneficial to evaluating Chinese medicine mechanism

**Fig. 3:**
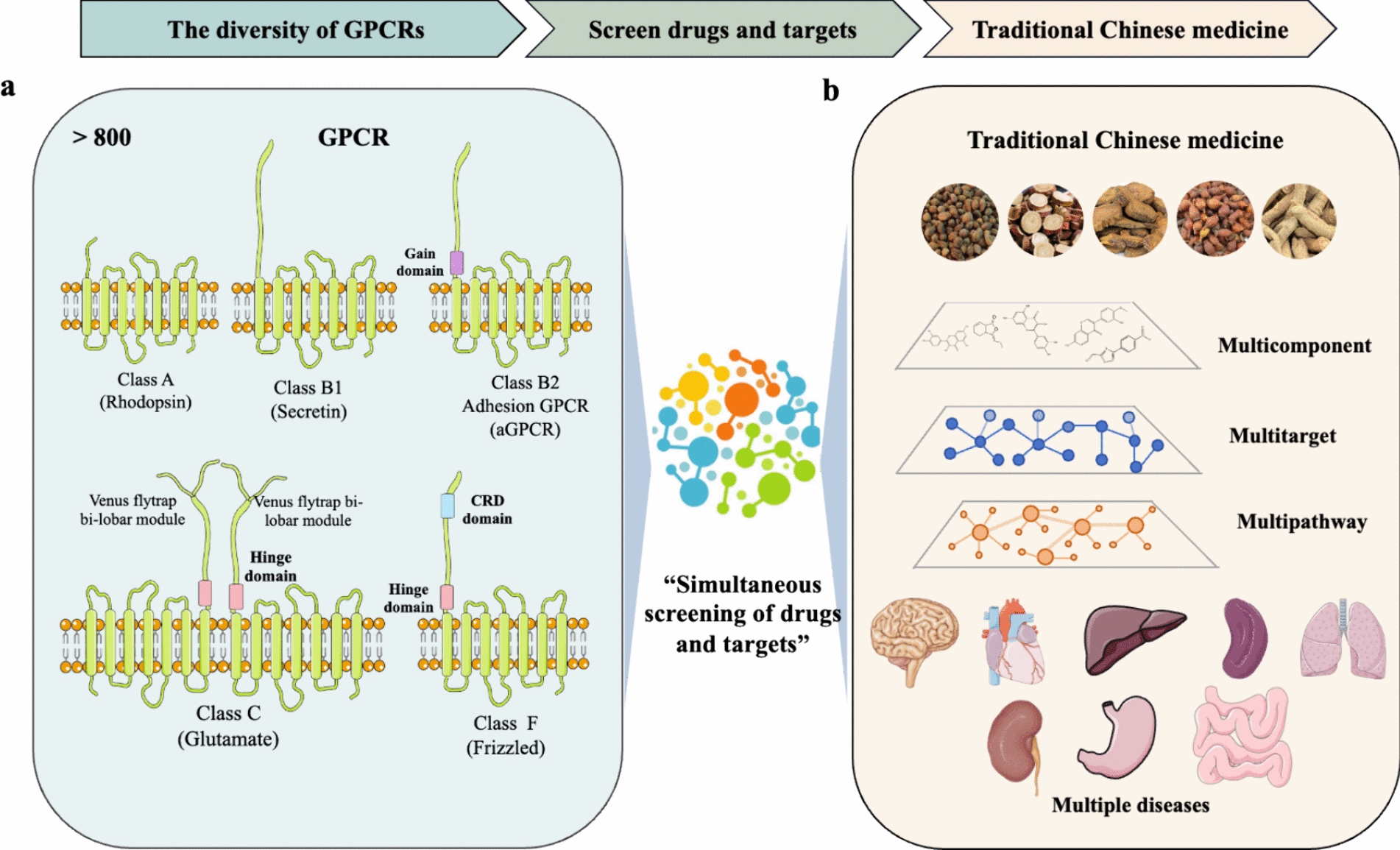
TCM and GPCRs, elucidating the theoretical basis and biological mechanism of TCM. **a** The diversity of GPCRs; **b** The characteristics of TCM and diseases

TCM often consists of various complex compounds that may act through multiple targets. Similarly, GPCRs, which constitute a large class of receptors, exhibit high structural and functional diversity. Therefore, the multiple components, targets, and pathways of TCM are consistent with the diversity of GPCRs, offering good prospects for TCM research (Fig. 3).

### Diversity of GPCRs is consistent with the multi-component and multi-target of TCM

GPCRs bind to various exogenous signalling molecules and triggering complicated intracellular signal transduction pathways [56]. This diversity is evident in the structural variances, activity characteristics and tissue- and cell-specific expression of GPCR subtypes [57]. For example, intestinal Takeda G protein-coupled receptor 5 (TGR5) promotes the secretion of glucagon-like peptide-1 (GLP-1) [58] and regulates blood sugar by acting on intestinal L cells. Additionally, TGR5 in adipocytes enhances brown adipose tissue function and induces white adipose tissue browning by regulating the expression of genes associated with glucose, fatty acid, and cholesterol homeostasis [59]. The activation of GPR35 can induce ATP synthase dimerization, reducing ATP loss during ischemia and preventing cerebral ischemia injury [60]. However, GPR35 serves as an early marker of heart failure because it is upregulated in the myocardial tissues of patients with heart failure [61]. GPR35 knockout can relieve hypertension induced by angiotensin II [62] and deoxycorticosterone acetate [63]. These studies demonstrated the complexity and functional diversity of GPCRs.

TCM often comprises complex compounds extracted from various natural plants, possessing diverse biological activities, and affecting multiple targets [64]. In contrast to the single-molecule targeted therapy of Western medicine, Chinese medicine emphasises overall regulation, achieving coordinated cellular and organ functions through the actions of multiple components and targets [65]. *Rhodiola crenulate* and its main component, salidroside, can prevent and treat brain injury at high altitudes through the ‘brain–lung’ axis [66]. Berberine from *Coptis Chinensis* can act on EIF2AK2, nucleic acids, gut microbiota and MAPK and exert anti-inflammatory pharmacological effects [67]. Notably, berberine can improve rat kidney injury caused by G protein-coupled receptor kinases [68]. Furthermore, bitter, sweet and olfactory receptors are also GPCRs [69]. Xuanfeibaidu granules can inhibit COVID-19 through ACE2 [65, 70, 71]. Verbenalin derived from *Verbena* can improve acute lung injury by targeting GPR18 [72]. These findings demonstrated the consistency between GPCRs and TCM characteristics, indicating that many TCM compounds exert pharmacological effects through GPCRs or GPCR-mediated signalling pathways.

### Characteristics of TCM and current multi-target drug development

Drug development is based on the idea ‘one disease, one target, one molecule’. However, the pathogenesis of most diseases is complex and diverse, and even the symptoms of the same disease vary. Consequently, drugs that target a single target are often struggling to meet treatment needs [73]. Initially, researchers believed that artemisinin targets only sarcoplasmic-endoplasmic reticulum calcium ATPase (SERCA) to combat malaria [74]; however, domestic researchers later discovered that artemisinin interacts with 124 targets [75], including GPCRs. These interactions provide the molecular basis for the multi-component and multi-target characteristics of TCM. A deep understanding of the interactions between active ingredients and GPCRs in Chinese herbs can provide insights for discovering novel and precise drug targets.

In contrast to a single drug that targets a single GPCR, TCM and herbal compounds often contain multiple components that may simultaneously affect multiple GPCR subtypes. This holistic regulation aligns more closely with the concept of the ‘holistic view’ of TCM, that is, the comprehensive treatment of diseases through multi-pathway and multi-level intervention [76]. For instance, Qingre Jiangzhuo prescription, a heat-clearing and detoxification compound, may regulate immune and inflammatory responses by targeting GPR119 and GLP-1R [77]. Associating GPCRs with TCM can enhance the efficiency of drug research and development. Understanding the multi-component and multi-target characteristics of TCM can facilitate the design and screening of candidate drug compounds with specificity and reduces the time and cost of drug research and development.

## GPCRs help in the discovery of new targeted TCM or drugs

**Fig. 4:**
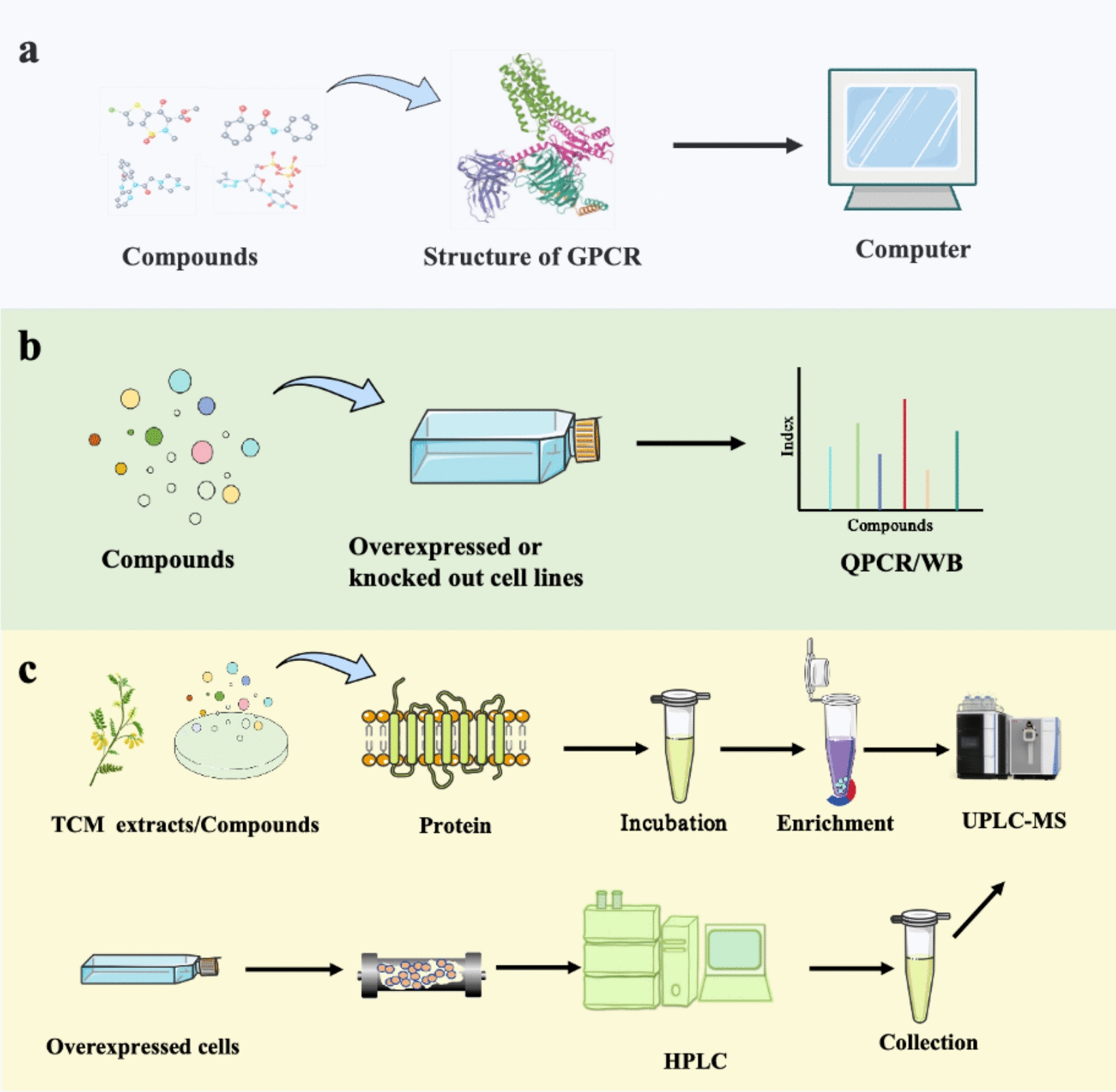
Commonly used GPCRs screening methods. **a** Virtual filtering based on structure; **b** HTS; **c** screening techniques based on proteins and small molecules

Several screening methods for GPCRs have been proposed, including high-throughput screening (HTS), structure-based virtual screening, protein–small molecule interaction-based affinity mass spectrometry and cell membrane chromatography (CMC; Fig. 4). These methods offer potential compounds for discovering novel drugs. For instance, anti-allergic asthma lead compounds were explored through Mas-related G protein-coupled receptor-X2 (MrgX2) CMC, targeting the mast cell MRGPRX2 [78]; natural products, such as diamine, shikotin and acetylshikotin, exhibited promising effects on asthma [79]. The three current screening methods are outlined below.

### High-throughput screening (HTS)

HTS is a critical technique in drug discovery and identification of potential candidates for pharmacological optimisation from compound libraries [80]. This technology integrates pharmacology, molecular biology, cell biology, computer technology and automatic control technology for rapid, efficient, micro-quantitative, automated, and scalable drug screening. Cell-based screening techniques include stable strain, CRISPR activation (CRISPRa) and CRISPR interference (CRISPRi) [81]. CRISPRa and CRISPRi have been utilised in screening autoimmune diseases and cancer, revealing genes that reprogram critical immune cell functions and informing the design of immunotherapies [82]. Additionally, the PRESTO-Salsa HTS platform with mRNA barcode technology as the core has successfully screened GPCR agonists [83]. Veozah [14], which was approved by the FDA in 2023, was obtained through HTS screening with subsequent structure optimisation.

### Virtual filtering based on structure

Structure-based virtual screening has been widely utilised to discover active molecules targeting various therapeutic targets [84]. Chemical and protein datasets with rich bioactivity data have been obtained [85]. Artificial intelligence, especially machine learning methods, including deep learning, has successfully utilised these datasets for the construction of score functions necessary for the virtual screening of targets with information about three-dimensional atomic structure [86]. These target-specific machine learning scoring functions are generally superior to traditional generality scoring functions, representing the latest advances in structure-based virtual screening techniques. For example, through structure-based virtual screening of 16 FDA-approved drugs against ROCK1, researchers have identified dasabuvir, which is a drug used to treat hepatitis C virus infection, as a potential drug for treating human enterovirus class A infection [87]. Our research group review clarified the flow of AI in GPCR ligand discovery. Using artificial intelligence, we have gained novel insights into complex TCM components and diverse GPCRs [88], thereby paving the way for the development of innovative therapies for a wide range of diseases.

### Screening techniques based on proteins and small molecules

The method based on protein–small molecule interaction has been successfully applied to screening ligands for various soluble drug target proteins. It is closely integrated with biochemical and cell function experiments, demonstrating high potential as a tool for discovering novel lead compounds and revealing novel targets within key signal transduction pathways [89]. Affinity mass spectrometry can efficiently and rapidly screen 28 potential ligands from a pool of 20,000 compounds, leading to the discovery of three new adenosine A2A receptor antagonists [90]. Additionally, 12 small-molecule regulators of 5-HT_2C_R and several previously unknown structural ligands have been identified by screening 4333 compounds [91]. These newly discovered ligands affect appetite and alleviate obesity, and four novel structural agonists specifically targeting GLP-1R have been discovered [92]. These findings suggest that affinity mass spectrometry play a prominent role in the screening of natural active compounds for protein targeting and in the regulation of protein function through intracellular metabolites. CMC and affinity mass spectrometry share the same principle. Therefore, utilising protein–small molecule interaction to target and screen the potential ligands of GPCRs is crucial for the development of novel drugs and exploration of pharmacological mechanisms.

### Concept of ‘simultaneous screening of drugs and targets’

The research strategy of Western medicine is usually based on target screening. Referencing the above several screening methods combined with the characteristics of TCM and GPCRs, we proposed a new screening strategy, ‘Simultaneous screening of drugs and targets’. This approach entails the concurrent screening of drugs and their potential targets. In brief, (1) disease targets are obtained with animal disease models or clinical data. (2) Known targets (e.g., HER2 for breast cancer [93]) and potential compounds in TCM or Chinese medicine compounds are screened. For example, nuclear receptor PXR is the target of autoimmune hepatitis [94], and PXR is used as a target for screening potential ligands from compound libraries or TCM. The general steps are as follows: disease targets are obtained through disease databases or experiments for receptor identification. Based on the principle of receptor action, the indirect screening of drugs is conducted, and the effects of drugs on receptors are assessed according to subsequent changes in biological effects after receptor activation. The aim is to provide an optimal therapeutic effect or personalised drugs for individual patients [95]. Additionally, direct screening is carried out to determine the target of action and the pathway of action by detecting the ability of a receptor to bind with a drug, thereby reflecting their interaction [96]. Utilising our statistical compound library of TCM and our GPCR target library, we conducted many-to-many virtual screenings to delineate the range of compounds. Subsequently, the selection was refined by subjecting the stable transmutation strain library to HTS [97, 98] followed by in vivo and in vitro experiments for verifying the efficacy of drugs and their action targets.

## Conclusion

The TCM syndrome and TCM emphasises individual differences and holistic concepts, and the expression levels and activity of GPCRs can vary among individuals. A study of the relationship between GPCRs and TCM is helpful to the realisation of personalised medicine therapy. Detailed information about a patient’s TCM syndromes and the status of relevant GPCRs facilitates the selection of a suitable drug regimen. Integrating TCM with modern drug research and development will facilitate the modernisation of TCM. Exploring the pharmacological mechanisms of TCM and combining them with advanced biotechnological methods are essential to the development of safe and effective drugs and establishment of a robust foundation for the integration of TCM into modern medicine.

The utilisation of TCM is limited because of its complex composition and unclear mechanism of action. Many GPCR drugs suffer from low specificity and high toxic side effects, thus limiting their effectiveness. The unstable structure and low expression of GPCR targets impedes the isolation of GPCRs. Therefore, the establishment of a standardised and widely applicable GPCR drug screening platform is a crucial step towards the advancement and modernisation of TCM. We aim to develop a comprehensive screening platform and cell library for GPCRs to promote the development of TCM and facilitate further research on GPCRs.

In summary, TCM treats different diseases with the same origin and employs the same treatment for different diseases, analogous to the concept of ‘simultaneous screening of drugs and targets’. Moreover, GPCRs are closely associated with TCM, and the fundamental theory and biological mechanisms of TCM can be elucidated by investigating TCM syndromes through GPCRs. The multi-component and multi-target characteristics of TCM align with the diversity of GPCRs, providing robust support for TCM scientific research and novel drug development. Further exploration of the relationship between GPCRs and Chinese medicine holds significant promise for the advancement and modernisation of TCM, personalised treatment and drug research and development. In conclusion, multi-target drug development is aligned more with the needs of complex diseases and contemporary drug development.

## Data Availability

All data generated or analysed during this study are published article.

## Abbreviations

ATL: Adult T-cell leukaemia lymphoma
BC: Breast cancer
CB2R: Cannabinoid receptor 2
class A: Rhodopsin-like receptors
class B1: Secretin-like receptors
class B2/aGPCR: Adhesion-like receptor
class C: Glutamate-like receptors
class F: Frizzled-like
CMC: Cell membrane chromatography
CRISPRa: CRISPR activation
CRISPRi: CRISPR interference
CXCR: Chemokine receptor
FDA: Food and Drug Administration
FFAR4: Free fatty acid receptor 4
FPR1: Formyl peptide receptor 1
GCGR: Glucagon receptor
GLP-1R: Glucagon-like peptide-1 receptor
GPCRs: G protein-coupled receptors
GPR35: G protein-coupled receptor 35
GPR54: G protein-coupled receptor 54
GRKs: G protein-coupled receptor kinases
HTS: High-throughput screening
IL-1β: Interleukin 1β
KOR: Kappa opioid receptor
MrgX2: Mas-related G protein-coupled receptor-X2
NF-κB: Nuclear factor κB
PAR1: Protease-activating receptor 1
PTH1R: Parathyroid hormone type 1 receptor
SSTR: Somatostatin receptor
TCM: Traditional Chinese medicine
TNF-α: Tumour necrosis factor α
